# Plin5, a New Target in Diabetic Cardiomyopathy

**DOI:** 10.1155/2022/2122856

**Published:** 2022-04-25

**Authors:** Xiangning Cui, Jingwu Wang, Yang Zhang, Jianliang Wei, Yan Wang

**Affiliations:** ^1^Department of Cardiovascular, Guang'anmen Hospital, China Academy of Chinese Medical Sciences, Beijing 100053, China; ^2^Clinical Education Division, Shandong University of Traditional Chinese Medicine, Jinan, Shandong 250000, China; ^3^First Clinical Medical School, Shandong University of Traditional Chinese Medicine, Jinan, Shandong 250000, China; ^4^Cardiovascular Department, Affiliated Hospital of Shandong University of Traditional Chinese Medicine, Jinan, 250000 Shandong, China

## Abstract

Abnormal lipid accumulation is commonly observed in diabetic cardiomyopathy (DC), which can create a lipotoxic microenvironment and damage cardiomyocytes. Lipid toxicity is an important pathogenic factor due to abnormal lipid accumulation in DC. As a lipid droplet (LD) decomposition barrier, Plin5 can protect LDs from lipase decomposition and regulate lipid metabolism, which is involved in the occurrence and development of cardiovascular diseases. In recent years, studies have shown that Plin5 expression is involved in the pathogenesis of DC lipid toxicity, such as oxidative stress, mitochondrial dysfunction, endoplasmic reticulum (ER) stress, and insulin resistance (IR) and has become a key target of DC research. Therefore, understanding the relationship between Plin5 and DC progression as well as the mechanism of this process is crucial for developing new therapeutic approaches and exploring new therapeutic targets. This review is aimed at exploring the latest findings and roles of Plin5 in lipid metabolism and DC-related pathogenesis, to explore possible clinical intervention approaches.

## 1. Introduction

Diabetic cardiomyopathy (DC) is characterized by metabolic changes in the myocardium that promote a chronic dysfunction of muscle fibers, leading to myocardium remodeling and heart failure, independently of the presence of coronary artery diseases or hypertension [1]. The pathophysiology of DC involves metabolic disorders, myocardial fibrosis, myocardial cell apoptosis, microangiopathy, oxidative stress, inflammatory response, and structural and functional changes in cardiac mitochondria [2–5]. The pathogenesis and signaling pathways of DC are intricate, making it difficult to identify effective targets for intervention. Despite renin-angiotensin-aldosterone system (RAAS) inhibitors and *β*-receptor antagonists have been used to combat cardiomyocyte apoptosis and myocardial fibrosis in DC, the results are not satisfying. Understanding the pathogenesis of DC and finding novel and effective intervention targets can provide new directions for clinical treatments of DC.

There are two forms of fatty acid (FA) in cells: one is free fatty acid (FFA), and the other is triglycerides (TGs) and stored in LDs. Abnormal increase in plasma FFA level is a specific indicator of diabetes as well as an important causative factor for DC. Plasma FFAs enter cardiomyocytes in a concentration-dependent manner and competitively inhibit glucose uptake, thereby limiting the use of glucose in cardiomyocytes and increasing the uptake and utilization of FFAs [6]. Generally, cardiomyocytes absorb excessive FFAs and are initially oxidized in mitochondria to provide energy for cardiac contractions. Other unutilized FFAs are stored in cardiomyocyte LDs such as TG, hydrolyzed to FAs, and glycerol by adipose triglyceride lipase (ATGL) with hormone-sensitive lipase (HSL) when needed [7, 8]. However, when the uptake of FFAs by cardiomyocytes far exceeds their utilization capacity, excessive mitochondrial oxidation can induce pathological oxidative stress and generate plenty of reactive oxygen species (ROS), which in turn damage mitochondrial structures and functions and aggravate impaired utilization of FFAs. Meanwhile, the accumulation of massive synthesized TG and its metabolic intermediate diacylglycerol (DG) in the myocardium will cause myocardial lipid toxicity [9–11]. Excessive accumulation of lipids and lipid intermediates in cardiac myocytes underlies the cardiac lipotoxic microenvironment. Excessive lipid accumulation can lead to pathological oxidative stress in cardiomyocytes, ER stress, mitochondrial dysfunction, insulin resistance (IR), cell signaling disorder, cardiomyocyte apoptosis, myocardial hypertrophy and fibrosis, coronary microvascular dysfunction, and even cardiac systolic dysfunction. Experimental and clinical data suggest that the reduction of toxic lipids improves myocardial metabolism and function [12–16].

Perilipin, located on the surface of LDs, acts as a molecular switch in the regulation of lipid metabolism. Plin5 belongs to the Perilipin family protein (protein acyltransferase, PAT). Plin5 is also named myocardial lipid droplet protein (MLDP) because it is highly expressed in the heart [17–19]. The increase of LDs in cardiomyocytes is the characterization of DC, which is consistent with the expression of Plin5 [20, 21]. Plin5 can promote the accumulation of myocardial lipid and alleviate cardiac lipotoxic injury [22–26]. A large number of experimental data have confirmed Plin5 is the key regulator of lipid metabolism [27, 28] and regulates metabolic procedures [29, 30]. Plin5 can inhibit ER stress, IR, oxidative stress, inflammatory and protect mitochondrial function, thus achieving a reduction in the level of cellular autophagy and apoptosis and protecting cardiomyocytes [31–36]. Evidence supports the direct role of Plin5 in the regulation of DC metabolic disorders and its pathogenesis. Therefore, accurate understanding of the regulatory mechanisms of Plin5 in DC pathogenesis will help to identify new targets.

### 1.1. An Overview of Plin5 Transcriptional Regulation and Interactions

Plin5 is a protein composed of 463 residues located on human chromosome 19 [22] that regulates transcription in the nucleus and located in the cytoplasm of mitochondrial, ER, and LD [17, 22, 37, 38]. Plin5 is mainly localized on the surface of LDs where it regulates the synthesis and catabolism of LDs through phosphorylation/nonphosphorylation status to keep the balance of FFAs in the cytoplasm and in mitochondria, where it participates in FA oxidation processes to coordinate the level of oxidative stress and energy supply of the body [39, 40]. Plin5/PGC-1*α* regulates lipid metabolism in response to extracellular signals to promote the full utilization of FFAs and avoid the accumulation of lipid intermediate metabolites [41]. Transcription of Plin5 in the nucleus is regulated by several factors. An experiment in porcine kidney showed that the transcription factor CCAAT/enhancer-binding protein *α* (C/EBP*α*) binds to the promoter region of Plin5 and induces its expression under fasting [42]. It has also been found that Jun protooncogene (Jun), activated transcription factor (ATF)1, ATF3, and ATF4 can also bind Plin5 promoter and induce its expression [43]. Among them, PPAR-*α* plays a major role in Plin5 transcription in human body. PPAR-*α* belongs to the activated receptor family of peroxisome proliferators (PPAR), which can regulate Plin5 transcription because the first intron of the gene contains the regulatory element (PPRE) [22]. PPAR consists of three subfamilies: PPAR-*α*, PPAR-*β*, and PPAR-*γ* [44], of which PPAR-*α* is highly expressed in cardiomyocytes, modulates the expression of key components of FA absorption, encodes for key proteins in oxidative signaling pathways through transcription, and maintains homeostasis in cardiomyocyte metabolism [45]. Plin5 was found to be induced in the liver in a PPAR-*α*-dependent manner under fasting conditions [17, 22, 37]. PPAR-*α* agonists can induce the expression of Plin5 in liver, skeletal, cardiomyocytes, and white adipose tissue (WAT) [46]. Plin5 promotes lipolysis, mitochondrial biogenesis, and oxidative metabolism mainly depending on the SIRT1/PGC-1*α*/PPAR-*α* pathway [47–49]. SIRT1, a member of the sirtuin family of NAD+-dependent protein deacetylases [50], is activated in ATGL-catalyzed lipolysis. Deacetylation can promote PGC-1*α* activity, which is required for ATGL-mediated upregulation of PPAR-*α*/PGC-1*α* signaling [51–54]. Recent findings found overexpression of Plin5 increased the expression of SIRT1, PGC-1*α*, and PGC-1*α* target genes [41], suggesting that Plin5 may be positively correlated with the expression of SIRT1, PGC-1*α*, and PGC-1*α* target genes. Consistent with Plin5 overexpression data, knockdown of Plin5 resulted in catecholamine stimulated expression of PGC-1*α* target genes [41]. Though the apparent regulatory effect of Plin5 on SIRT1, SIRT1 activation is mediated by cAMP/PKA-dependent phosphorylation associated with increased cellular NAD+, rather than its interaction with Plin5 [41, 55, 56].

**Figure 1:**
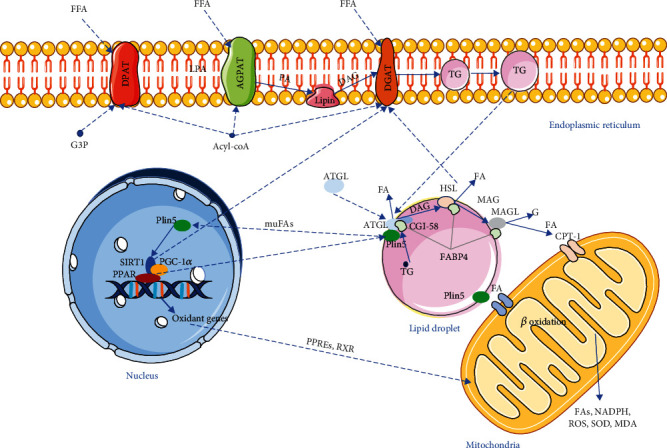
The process of lipid metabolism in DC. FFAs in cells synthesize TG under the action of Acyl-coA, DPAT, AGPAT, Lipin, and DGAT protein and store TG in LD. With the increase of TG synthesis, LD increases gradually and is separated from ER into cytoplasm to isolate FAs and reduce lipid toxicity. When stimulated by external stimuli, Plin5 recruits ATGL to LD, Plin5 is phosphorylated and inactivated, CGI-58 is released, ATGL and HSL are activated, and lipolysis is initiated. Under the action of corresponding proteases, TG is decomposed into FA, which is released into cytoplasm by FABP4 or directly into mitochondria through Plin5-mediated LD-mitochondrial contact. Meanwhile, Plin5 can carry muFAs into the nucleus. Plin5 binds to SIRT1 PGC-1*α* to form a complex that activates PPAR and oxidative gene expression, promoting lipolysis and mitochondrial *β*-oxidation.

As shown in Figure 1, Plin5 binds to ATGL and reduces the FAs generated by the lipolysis process, hence reducing the amount of FFAs in cells. ER-related proteins (such as DAPT, DGAPT, Lipin, and DGAT) bind FFAs under the action of Acyl-coA and convert them to TG and then storing them in LD, reducing the damage of cardiomyocyte lipotoxicity by isolating TG. What is more, LDs also can promote autophagy by storing neutral lipids to support Plin5-dependent autophagic membrane formation [57]. Therefore, Plin5 is indirectly involved in the process of cellular autophagy by cooperating with LD in regulating FA. As the conversion into TG molecules gradually increases, their size gradually increases [58]. When TG grows to a certain extent, it is released from the ER into the cytoplasm to form LD. When hypoxia, ischemia, or pressure overload occurs, FA is mobilized and stored in the LDs, and Plin5 is phosphorylated in a PKA-dependent manner and translocated from the LD to the nucleus to activate SIRT1, forming a complex with SIRT1 and PGC-1*α* to activate PGC-1*α*/PPAR-*α* [41, 47, 48, 59] that promotes TG catabolism and mitochondrial FA oxidation. Phosphorylated Plin5 is released, binds to ATGL, and activates CGI-58 to drive the lipolysis process [27, 28, 60]. Under the action of Plin5 together with ATGL, HSL, and monoacylglycerol lipase (MAGL), TG is finally decomposed into three FAs and one glycerol molecule and released into the cytoplasm after a series of biochemical reactions [27, 35, 61]. FAs can be efficiently transported to mitochondria for *β*-oxidation via carnitine lipid acyltransferase (CPT-1, *β*-rate limiting enzyme) or plin5-mediated LD-mitochondrial contacting [62, 63], thereby reducing lipotoxicity caused by excessive accumulation of FFAs in cells.

Plin5, SIRT1, PGC-1*α*, and its promoter PPARs regulate the FA *β*-oxidation process in mitochondria, of which PGC-1*α* plays an important role in mitochondrial biogenesis and energy metabolism [64, 65]. In particular, the expression of PGC-1*α* is positively correlated with the expression of TCA cyclase, which promotes hypoplasia and leads to heart failure (HF) [66, 67]. Enhanced expression of Plin5 in failing heart may activate PGC-1*α* and improve TCA cyclase activity. In mitochondria, FA oxidation not only generates energy but also releases ROS. When FA overload leads to enhanced mitochondrial oxidation, excessive ROS are released into the cells causing myocardial damage. ROS are essential intracellular second messengers that play an important role in the regulation of inflammation, oxidative stress response, cell growth, and differentiation. Therefore, it can be inferred that Pin5 is involved in the regulation of lipolysis and FA metabolism, as well as in the corresponding pathological processes such as cellular inflammation and oxidative stress.

Plin5 is highly concentrated and expressed in cardiomyocytes, skeletal muscle, liver, and other oxidized tissues [17, 22, 37], and a small part is distributed in islet *β*-cells and liver stellate cells [68, 69]. In whole-body energy metabolism, Plin5 can protect muscle cells by promoting LD decomposition reducing internal fuel consumption and controlling FA flux to reduce lipotoxicity [24, 70]. It plays different roles in different tissues. For example, Plin5 can protect pancreatic *β*-cells and regulate lipid metabolism in mouse islet *β*-cells [32, 69], as well as regulate fasting induced IR and lipotoxic in muscle cells [23]. These results suggest that Plin5 expression is tissue-specific and can ameliorate pathological changes such as IR, lipotoxicity, and ell injury in different tissues through several pathways.

The expression of Plin5 was positively correlated with increased steatosis in cardiac, skeletal muscle, and liver tissue [30, 71–74] but negatively correlated with the expression of oxidative metabolism-related gene (such as PPAR-*α*, PGC-1*α*, and related genes), mitochondrial function indicators, and FA oxidation level in the heart [71, 72]. This difference may be related to the inherent ability of the normal adult heart to handle the TG rate and Plin5-independent FA flux, which is a characteristic of cardiac hypertrophy [75–77]. In Plin5 knockout mice, researchers monitored significant reductions in TG and LD content in their skeletal muscle, liver, and heart [24, 35, 70]. Plin5 deficiency is associated with increased accumulation of ceramides and IR in skeletal muscle [70]. In the liver, it is associated with PPAR-*α*-activated hepatocyte nonesterified FAs, increased ER stress markers, inflammation, and tissue damage [35]. In the heart, it can lead to an increase in FA oxidation associated with age-related cardiomyopathy [24], which verifies the important role of Plin5 in lipid metabolism.

In conclusion, although Plin5 has specific expression in different tissues, its basic role is major in regulating lipid metabolism. Plin5 can promote FA synthesis and storage of TG in LD to reduce lipid toxicity in physiological states of steatosis and its related oxidative stress, inflammation, IR, ER stress, and other pathological processes. Plin5 enhances the expression of lipids, accelerates lipolysis, and promotes lipid utilization during lipid increase and steatosis. Therefore, we can speculate that Plin5 may play a bidirectional role in lipid metabolism, while the deficiency of Plin5 reduces the synthesis of TG and LD in cells, and even promotes the appearance of cardiomyopathy related to lipid metabolism in the heart. Plin5 has great research value and development potential to improve DC pathological changes. A full understanding of DC pathological changes and pathogenesis is conducive to promoting future studies on Plin5 intervention in DC treatment.

## 2. The Role of Plin5 in Lipid Metabolism and DC

Obesity [78], IR [79, 80], dyslipidemia [81], and type 2 diabetes (T2DM) [82, 83] are basic risk factors for cardiovascular disease. These risk factors include imbalance of glucose, lipid metabolism, and changes in blood flow leading to heart remodeling [84, 85]. Myocardial remodeling is a disorder of myocardial cell structure and function caused by molecular biological and genetic changes under neurohumoral factors [86]. Cardiac remodeling can flexibly adjust energy supply and improve cardiac function during short periods of myocardial ischemia and hypoxia [87, 88]. However, long-term cardiac remodeling leads to cardiac cell death [89, 90], promoting the development of DC and ultimately developing heart failure (HF) [91].

In the early stages of diabetes, IR leads to metabolic changes in cardiomyocytes that increase FA intake and *β*-oxidation, to maintain adequate levels of ATP production. However, when the FFAs being ingested far exceed those need for *β*-oxidation, intracellular lipid accumulation will lead to lipid toxicity [92]. It is well known that patients with obese diabetes show more significant visceral fat deposition. Diabetes is associated with significantly abnormal cardiac structural changes in steatosis [93]. However, large lipid accumulation can also cause cardiac dysfunction. In fulminant type 1 diabetes (T1DM) patients complicated with cardiac shock, the electron microscope showed large amounts of LDs and a wide range of inflammatory cell infiltration in the myocardial cells, and cardiac ultrasound showed the heart appeared to have serious cardiac insufficiency [20]. TG accumulation can increase free radical sources, which can induce oxidative stress and trigger lipid peroxidation [94]. This is accompanied by the release of proinflammatory cytokines, mitochondrial damage, and ER stress, aggravating myocardial injury. Modern pharmacological reports believe that with the progression of cardiac hypertrophy, cardiac energy metabolism disorder, and limited use of FA, lipid accumulation will aggravate the occurrence of cardiac hypertrophy and HF [95, 96]. Therefore, with the increase of glucose and lipid metabolism disorder, the damage to the heart also gets worse constantly.

Steatosis is usually associated with increased lipolysis and IR, leading to increased FFA levels. In physiological state, cardiomyocytes preferentially use FAs in a concentration-dependent manner in many cardiac energy substrates such as ketone body, lactate, FAs, and glucose [97]. In diabetes, impaired insulin signaling pathways lead to increased lipolysis and increased levels of FFAs in the body, resulting in increased utilization of FAs [98]. Excessive FFAs can produce lipid toxicity to cardiomyocytes. Despite high glucose levels, heart muscle cells from diabetic patients use relatively low level of glucose [99, 100]. Experimental studies [101] found that increased glucose transport in the myocardium after the short-term onset of diabetes would aggravate mitochondrial oxidative dysfunction, thus reducing the glucose utilization rate in the myocardium. Part of the accumulated glucose is converted into lipids and stored in LD, while the other part of the glucose that is too late to be decomposed generates a large amount of ROS to damage *β*-cells, causing glucotoxicity and aggravating islet cell damage. The appearance of lipid toxicity, glucotoxicity, and their interaction will further aggravate cardiomyocyte damage in DC.

Plin5 is a key protein in cardiac remodeling, which is involved in intracellular lipid storage and lipid oxidation. LDS can regulate FFA metabolism to maintain lipid homeostasis and endothelial cell survival in diabetic CMECs by balancing the expression status of Plin5 [102]. It can also regulate the exposure process of lipid metabolism in the cytoplasm to reduce the lipotoxicity by mechanisms such as antioxidative stress [24, 103]. Plin5 deficiency increases complexes I and III, the dominant ROS production sites in the mitochondrial respiratory chain, activates PPAR-*α*, promotes mitochondrial proliferation, and increases the rate of lipolysis, thereby exacerbating stress overload-induced cardiac remodeling [104]. T2DM can lead to changes in fat metabolism, increase liposolysis, elevate levels of circulating FAs, and enhance peripheral tissue uptake. Myocardial remodeling usually occurs in patients with T2DM [105, 106]. In hyperfree fatty acidemia T2DM mice, Plin5 deficiency leads to a reduction in capillary numbers and exacerbates microvascular endothelial injury and myocardial diastolic dysfunction [102, 107]. CM-Plin5 deficiency reduces ATGL and HSL-mediated lipolysis [72]. In vitro experiments have shown that Plin5^−/−^ cardiomyocytes maintain higher glycogen content and exhibit better hypoxia tolerance and less LD storage compared to Plin5^+/+^ [108], suggesting that Plin5 can regulate the balance of glycolipid metabolism and protect ischemic myocardium. However, CM-Plin5 overexpression causes cardiac steatosis, increased heart weight, left ventricular hypertrophy, and mild cardiac dysfunction in mice [71, 72]. In conclusion, the expression status of Plin5 determines whether its regulatory effect on lipid metabolism is positive or destructive to the heart.

Because the effect of diabetes on the body is broad, DC is not only the influence of diabetes on the heart, but it also affects the liver, pancreas, vascular, WAT, and skeletal muscle of physiological and pathological changes. Therefore, a comprehensive understanding of Plin5 in different organizations helps us to explore the value of Plin5 in DC diagnosis and treatment.  Table 1 shows the known physiological effects and related mechanisms of Plin5 on the heart, liver, pancreas, vessels, skeletal muscle, etc. It can be seen from Table 1 that Plin5-related mechanisms in DC include primarily oxidative stress, inflammatory response, IR, ER stress, apoptosis, and mitochondrial damage. FA content and conversion play a key role in this process.

## 3. The Cellular Processes of Plin5 in DC

**Figure 2:**
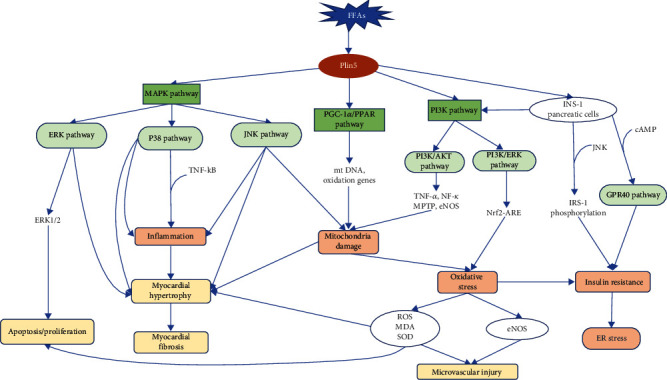
The lipid metabolism mechanism of Plin5 in DC. In oxidized tissues and adipocytes, Plin5 mainly regulates fatty acid metabolism through the MAPK pathway, PGC-1 *α*/PPAR pathway, and PI3K pathway. In pancreatic cells, Plin5 interferes with the PI3K pathway, GPR40 signaling pathway, and IRS-1 phosphorylation pathway to achieve its effect on pancreatic cells. When FA metabolism is disturbed, Plin5 can reduce the microvascular damage, myocardial hypertrophy, myocardial fibrosis, apoptosis/proliferation caused by FA by coordinating the mechanism of mitochondrial damage, oxidative stress, inflammation, insulin resistance, and ER stress.

Early stages of DC are usually asymptomatic and manifest as left ventricular hypertrophy, fibrosis, and abnormal cell signaling, characterized by abnormal atrial filling and reduced left ventricular diastolic function [99, 115, 116]. Underlying pathological factors include hyperglycemia, IR, elevated FFA levels, inflammation, ER stress, and mitochondrial dysfunction [99, 115]. Symptomatic left ventricular enlargement and HF occur after systolic dysfunction [117, 118]. Reduced cardiac diastolic function was observed in both T1DM and T2DM [119], but enhanced cellular autophagy but not hypertrophic cardiomyocytes was observed in T1DM mice [120], whereas cardiac hypertrophy but suppressed cellular autophagy was observed in T2DM mice [119]. This difference may be related to the different diabetic phenotypes and underlying mechanisms [121], but both related with pathological alterations in cardiac cells [3]. Further understanding of its pathological mechanism is helpful to explore potential targets of Plin5 in the DC diagnosis and treatment. Therefore, this section reviews the pathological changes of cardiomyocyte apoptosis, cellular autophagy, myocardial hypertrophy and fibrosis in DC, and microvascular injury due to damage of the microvascular endothelium between cardiomyocytes along with the associated pathogenesis of oxidative stress, mitochondrial dysfunction, inflammation, ER stress, and IR (Figure 2).

### 3.1. Apoptosis/Autophagy

Autophagy is an important component of cardiomyocyte homeostasis that increases cell survival after cellular stress and starvation [122]. In the presence of nutrient deficiency, the induction of autophagy provides cells with the opportunity to reuse their own components for energy [123]. However, under certain circumstances, autophagy not only protects cells from death but also mediates cell death. Autophagic cell death occurs if autophagy destroys cytoplasm and organelles beyond a certain threshold [124]. Autophagy is mainly mediated by Atg proteins [125], and apoptosis is mainly regulated by the bcl-2 family of proteins and the cysteine family of enzymes [126]. Although autophagy and apoptosis proceed through different signaling pathways, many studies have shown that activation of the PI3K/Akt signaling pathway can inhibit apoptosis and excessive autophagy [127, 128].

Activated Akt is a downstream effector of PI3K and inhibits apoptosis by regulating multiple targets such as mitochondrial permeability transition pore (MPTP), tumor necrosis factor *α* (TNF-*α*), endothelial nitric oxide synthase (eNOS), bcl-2 family proteins, and NF-*κ*B24. Experimental studies showed that PI3K and Akt phosphorylation levels in heart tissue from Plin5-deficient mice were reduced [21], suggesting that Plin5 may activate the PI3K/Akt signaling pathway to inhibit autophagy/apoptosis. Other possible mechanisms include that oxidative stress can activate the intrinsic apoptotic pathway of pancreatic *β*-cells [129], ER stress can lead to Ca^2+^ disorder and promote autophagy/apoptosis [130, 131], mitochondrial dysfunction exacerbates oxidative stress levels activating the apoptosis/autophagy-related pathway [4, 132, 133], and IR can activate GPR40 signaling pathway and JNK pathway aggravating lipid metabolism disorder [68, 134, 135].

#### 3.1.1. Oxidative Stress

When DC develops to the stage of cardiac hypertrophy, the heart uses glucose instead of FAs as its main energy source to reduce oxygen consumption and promote the production of ROS [136–138]. At the same time, lipid accumulation increases, and glucose utilization is limited [95, 96]. Excessive cytoplasmic FFAs can stimulate *β*-oxidation, release excessive ROS, and aggravate oxidative stress [35]. Medium anti and high concentrations of ROS can induce cell apoptosis and even lead to cell necrosis through oxidative stress [10, 60]. Excess ROS can exacerbate myocardial remodeling through DNA damage, protein oxidation, matrix metalloproteinase activation, and lipid peroxidation, leading to cellular dysfunction and cardiomyocyte apoptosis [139].

Plin5 disrupts the interaction between ATGL and its coenzyme *α*-*β* hydrolase domain 5 (ABHD5/CGI-58), inhibiting LD lipolysis and excessive FA oxidation [35, 104, thus reducing the production of ROS and oxidative stress levels. Plin5 expression has different effects on oxidative stress expression. Studies have shown that overexpression of Plin5 can enhance antioxidative gene expression in skeletal muscle [30]. While Plin5 deficiency can lead to proliferation of myocardial mitochondria, promote mitochondrial oxidation, increase ROS and malondialdehyde (MDA) levels, and decrease superoxide dismutase (SOD) activity [21], aggravating oxidative stress. ROS and MDA are the products of lipid peroxidation and are induced by oxygen free radicals. ROS and MDA can reflect the degree of cell damage, while SOD can remove superoxide anion and protect cells from damage. Therefore, Plin5 deficiency aggravates ROS-mediated cardiac injury [24]. Plin5 has been shown to be negatively correlated with oxidative stress in lipid overload-induced mouse tissues [24, 110, 140], and Plin5 has a significant antioxidant effect on oxidative stress [32].

In cardiovascular and liver, Plin5 is involved in the regulation of oxidative stress through PI3K/Akt or MAPK pathways (P38, ERK, and JNK) [21, 140–143] and is also an upstream regulator of Nrf2 in pancreatic *β*-cells [143, 144]. PI3K/Akt is an important antiapoptotic/proliferation signaling pathway [145], which can regulate it by regulating mitochondrial proliferation and apoptosis. The MAPK pathway is an important pathway for mitogen-activated protein kinases (MAPKs) to coordinate cell metabolism and apoptosis, recognizing and responding to extracellular stimuli [146]. The most extensively studied MAPK pathways are ERK1/2, JNKs, and P38 kinases [146–148]. The ERK1/2 signaling pathway is a key regulator of cell proliferation [147]. The P38 regulates the expression of several cytokine transcription factors and cell surface receptors [148], and its activity is crucial for immune inflammation. JNKs can activate mitochondrial apoptosis pathway [149] and inhibit insulin receptor signal transduction, leading to IR [150–152]. Meanwhile, obesity-induced lipid toxic stress can activate JNK pathway [153–155], resulting in a vicious cycle of lipid metabolism in vivo. Experimental studies have found that Plin5 can increase the activity of the PI3K/Akt and ERK pathways and reduce the activity of the P38 and JNK pathways [32], thus achieving the effect of antioxidant improvement of IR and reduction of cell stress and apoptosis.

Plin5 reduces oxidative damage related to lipid toxicity by activating PI3K/ERK-mediated Nrf2-ARE signaling pathway in INS-1 pancreatic cells [32]. Nrf2 is a major regulator of oxidative stress in cells [144]. Oxidative stress activates Nrf2 in pancreatic *β*-cells, which promotes the expression of antioxidant enzymes by binding to ARE in promoter region [144, 156], thus promoting antioxidant capacity, improving the function and survival rate of *β*-cell.

#### 3.1.2. Mitochondrial Dysfunction

Mitochondria typically occupy approximately 20% to 30% of the total cell volume of cardiomyocytes [99]. Typically, mitochondrial oxidative phosphorylation produces more than 95% of ATP, with the remaining 5% produced by the glucose and lactate tricarboxylic acid cycles [157]. However, in an environment of hyperglycemia, IR, and hypertriglyceridemia, cardiomyocytes have a reduced ability to utilize glucose, with FFAs as the main source of energy [100].

The high glucose environment in DC was shown to inhibit the activity of AMPK-activated protein kinase *α*2 and increase the expression of human protein (Fundc1) from the outer mitochondrial membrane [158, 159], promoting the formation of the mitochondria-associated endoplasmic reticulum (MAM) in diabetes, leading to increased mitochondrial Ca^2+^, mitochondrial rupture, mitochondrial dysfunction, and myocardial dysfunction [4, 132, 133]. Plin5 is involved in mitochondrial fission, which requires phosphorylation of mitochondrial fission factor (Mff) to recruit dynamin-related protein 1 (Drp1) [160]. Reduced amount of phosphorylated Mff in transgenic mouse cardiomyocytes with the Plin5 gene encoding serine-155 to alanine exchange implies reduced recruitment and fission of mitochondrial Drp1, which may protect against lipotoxic-induced cardiac dysfunction [161].

PGC-1*α* synergistically activates PPAR and plays a key role in the regulation of mitochondrial FA *β*-oxidation and mitochondrial oxidative phosphorylation [17, 24, 37, 162]. PPAR and PGC-1*α* regulate the expression of Piln5, and its miRNA and protein are highly enriched in oxidized tissues such as heart [37]. In the absence of Plin5, the expression of PPAR-*α* protein and miRNA was significantly increased in the liver [17], and the levels of PPAR-*α* and PGC-1*α* were significantly upregulated in cardiac myocytes [104]. PPAR may be associated with peroxidase proliferator reaction elements (PPREs) and retinoic acid X receptor (RXR) binding, thus increasing FA oxidation capacity. PPAR-*α* is a direct regulator of Plin5 [33], while the induction of Plin5 expression by PPAR*β*/*δ* and/or PPAR-*γ* activation is indirect. The specific regulation mechanism still needs further study. SIRT1 plays a key role in sensing intracellular REDOX (NAD), coordinating cell functions [52–54], that can promote LD catabolic metabolism and is responsible for downstream FA metabolic pathways [59]. SIRT1 deacetylates PGC-1*α* and promotes its interaction with PPAR-*α* [59], increasing PGC-1*α*/PPAR-*α* activity [41].

The lipolysis process is significantly activated in response to increased energy requirements. Plin5 promotes mitochondrial biogenesis and oxidative metabolism through SIRT1/PGC-1*α*/PPAR-*α*-dependent pathways. Recent experiments have shown that Plin5 preferentially binds LD-derived monounsaturated fatty acids (MUFAs) after activation and phosphorylation of PKA, transports it to the nucleus under cAMP/PKA-mediated lipolytic stimulation, activating SIRT1 and forming a complex with SIRT1 and PCG-1*α* to activate the lipolysis process [41, 59], and promotes mitochondrial biogenesis in BAT and muscle. In cells and animal models, MUFA enhances PGC-1*α*/PPAR-*α* signaling and promotes oxidative metabolism in a SIRT1-dependent manner. When Plin5 is deficient, FFAs can also increase transcription of PPAR [41, 163], leading to upregulation of PPAR-*α* and PGC-1*α* levels. At the same time, the content of mitochondrial DNA (mtDNA) and the number of mitochondria in cardiomyocytes are significantly increased, thus stimulating mitochondrial proliferation [104] and oxidation-related gene transcription [41], increasing the degree of oxidative stress.

#### 3.1.3. Endoplasmic Reticulum Stress

Insufficient autophagy, excess or restricted nutrition, excessive oxidative stress, and inflammation can disrupt ER homeostasis, leading to the accumulation of unfolded or misfolded proteins in the ER compartment, called ER stress, which activates the unfolded protein response (UPR) [164]. Under physiological conditions, the UPR helps cells adapt to ER stress and regulates the cellular life state [165]. In acute ER stress, the UPR is activated to restore ER protein folding homeostasis [166]. If ER stress is chronic or severe, UPR activation can lead to Ca^2 +^ disorder [130, 131] and promote cell survival or cell death [167, 168]. ER stress reveals a perturbation of ER function, which further initiates UPR and ER stress-related autophagy to reestablish ER homeostasis; otherwise, unresolved ER stress inevitably leads to cell death through induction of apoptosis [169, 170]. Indeed, the crosstalk between UPR and autophagy or apoptosis determines cell fate and is controlled by multiple signaling pathways. The way in which lipids cause ER stress is unclear, but there is growing evidence that this conserved response plays an important role in maintaining metabolism and lipid homeostasis.

LD accumulation is related to ER stress in the yeast and mammalian liver [171, 172], which stimulates the formation and accumulation of LD [173], and promotes the appearance of lipid toxicity. ER stress is an important factor in the generation of pancreatic *β*-cell lipid toxicity [174, 175]. In pancreatic *β*-cells, Plin5 alleviates ER stress induced by overnutrition [33]. ER stress is associated with IR [176], which is thought to be an imbalance between protein folding capacity and increased demand for insulin production and secretion [164, 177].

Plin5 acts as a protective factor against lipid toxicity in the liver and skeletal muscle and regulates ER stress [35, 70, 110, 178]. Studies have shown that Plin5 overexpression can slow down lipolysis and FA oxidation under basic conditions [178]. It can significantly promote FA oxidation in skeletal muscle under stimulation of starvation or activated PKA [178]. It is common sense to speculate that Plin5 may promote LD formation and inhibit ER stress under underlying conditions. However, studies have found that loss of Plin5 in skeletal muscle can reduce ER stress [179], and there is no significant difference in ER stress in the heart even if Plin5 is completely lost [179]. The regulation mode of Plin5 in ER stress needs further study.

#### 3.1.4. Insulin Resistance and Signal Conduction

Insulin resistance (IR) is a reduction in glucose utilization by target organs in response to insulin [180] and is an important risk factor for cardiovascular disease [180, 181]. Inflammation, mitochondrial dysfunction, hyperinsulinemia, and hyperlipidemia can all induce IR [180], with high concentrations of circulating FFA-induced lipotoxicity being a key mechanism of IR [182]. In animal studies, mice were found to develop myocardial IR because of a high-fat diet, characterized by downregulation of IR activity, reduced Akt signaling, and a shift from glucose utilization to fatty acid utilization [183]. The role of autophagy in insulin resistance has been controversial [184–186]. Recent studies have found that in a rat H9c2 cardiomyocyte IR model [187], cardiomyocytes show hyperactivation of autophagy and increased apoptosis with increasing insulin resistance, and the PI3K/Akt/mTOR pathway is involved in this process. The PI3K/Akt/mTOR pathway is the link between insulin resistance and autophagy. The two major insulin receptor substrates, IRS-1 and IRS-2, activate the PI3K/Akt pathway [188], while the PI3K/Akt/mTOR pathway inhibits autophagy when activated [189]. It has been shown that Plin5 can inhibit autophagy by regulating the activation of mTOR that can be activated through the PI3K/Akt pathway [190].

Plin5^−/−^ mouse models have demonstrated that Plin5 can improve IR in the skeletal muscle of the insulin sensitive liver with glucose tolerance [70], and the specific pathway mechanism is still being explored. According to previous studies, Plin5 also can improve insulin resistance by mediating the JNK pathway. FFA is an effective activator of JNK [191–194], and FFA induces insulin resistance by inhibiting the serine phosphorylation of IRS-1 [134, 135]. Biochemical studies have confirmed that IRS-1 phosphorylation is the target of JNK-mediated insulin resistance [150, 153]. The activity of Jun's N-terminal kinase JNK is primarily provided by JNK1, and when JNK1 is lost, its inhibition of IRS-1 phosphorylation can be reduced and the ability to transduce insulin receptor signals can be improved to some extent [153]. Studies have shown that the loss of JNK1 can promote the reduction of obesity, the significant improvement in insulin sensitivity, and the enhancement of insulin [153]. However, whether IRS-1 phosphorylation is the only target of JNK-mediated insulin resistance remains to be further studied. On the one hand, Plin5 can accelerate the utilization of FFAs by lipid-mitochondrial contact [39] or reduce the activation effect of FFAs on JNK by isolating FFAs [24]. On the other hand, Plin5 can reduce the activity of the JNK pathway and reduce the inhibition of IRS-1, thus achieving the effect of improving insulin resistance.

Plin5 can also regulate lipid metabolism and promote postprandial insulin secretion in a manner dependent on cAMP and the G protein-coupled receptor 40 (GPR40) [68]. *β*-Cell dysfunction is a basic defect of diabetes [195], leading to insufficient basal insulin secretion and long-term poor control of postprandial hyperglycemia, leading to macrovascular lesions. FA promotes postprandial insulin secretion by acutely increasing insulin release [196, 197] and activates GPR40 to produce intracellular FA metabolites [198]. GPR40 is a highly expressed FA receptor on the cell surface [199]. GPR40^−/−^ mice showed that approximately 50% of FA-mediated enhancement of insulin secretion is dependent on GPR40 [200]. It was found that the destruction of GPR40 in mice reduced the induction of insulin secretion by FAs [200]. In contrast, transgenic overexpression of GPR40 in mice improved glucose-stimulated insulin secretion in wild-type and diabetic mice [201]. This suggests that GPR40 plays an important role in regulating insulin secretion. Plin5 may engage in a cross-dialogue with the GPR40 signaling pathway in extracellular secretion of FA or in collaboration with the GPR40 signaling pathway in cells [68]. Intracellular lipid metabolism and GPR40 signaling are intertwined rather than operating independently. Plin5 also relies on CAMP-activated protein kinase (PKA) to mediate increased lipolysis, which may be sufficient to alter the structure of intracellular lipid metabolites and thus participate in the regulation of insulin secretion [68].

#### 3.1.5. Inflammation

Autophagy is a key link in the autonomous control of inflammation by cells [202]. Uncontrolled inflammation is one of the main factors in the pathogenesis of cardiomyopathy [203]. Organelle autophagy plays an important role in cell protection; for example, activation of mitochondrial autophagy can protect cardiomyocytes from lethal inflammation by promoting an anti-inflammatory response, inhibiting cardiomyocyte loss, and promoting the protective effects of tissue remodeling and fibrosis [204, 205]. Research finds that Plin5 promotes autophagy and prevents FA-induced inflammation through SIRT1 signaling during fasting [206], while Plin5 deletion has also been found to reduce the inflammatory response in liver cells [178]. P38 is the second prototype member of the MAPK-related pathway in mammalian cells [207, 208], which can reduce the activity of the inflammation pathway [32, 209]. Theoretically, Plin5 can restrain inflammatory immunity through this pathway, but the mechanisms between Plin5 and inflammation are still largely unknown.

### 3.2. Myocardial Hypertrophy and Fibrosis

Plin5 deficiency increases oxidative load, which exacerbates stress overload-induced cardiomyocyte hypertrophy [104]. It is well known that ROS induce cardiac hypertrophy and cardiac dysfunction through oxidative damage and/or abnormal redox signaling [21, 210]. In a mouse model of transverse aortic constriction- (TAC-) induced cardiac hypertrophy and heart failure [104], Plin5-deficient mice had an enlarged cardiomyocyte cross-section compared to wild mice after TAC, and their lipid content in the myocardium was reduced approximately three times more than that of wild-type mice. Moreover, in Plin5-deficient mice, a significant increase in mitochondrial number and mitochondrial DNA content was observed, with significantly higher levels of MDA and ROS and significantly lower SOD activity. This suggests that Plin5 effectively reduces lipid content [24], stimulates mitochondrial proliferation, and exacerbates myocardial oxidative stress [21].

Activation of PPAR by PGC-1*α* is known to play a key role in regulating fatty acid mitochondrial *β*-oxidation as well as core genes involved in mitochondrial oxidative phosphorylation [17, 24]. mRNA levels of PPAR-*α* and PGC-1*α* were significantly increased in Plin5-deficient cardiomyocytes after TAC surgery [104], which may contribute to mitochondrial proliferation and increased levels of enzymes involved in oxidative phosphorylation elevated levels. Taken together, the increased mRNA expression of PPAR-*α*/PGC-1*α* in the absence of Plin5 stimulates an increase in mitochondrial DNA content, promotes mitochondrial proliferation to increase oxidative levels, accelerates the utilization of FFAs in cells, and reduces lipid synthesis. As a result of increased oxidation levels, the released ROS in turn stimulate cellular hypertrophy.

Hyperglycemia, IR, and oxidative stress all can promote the expression of cardiomyocyte hypertrophy genes such as the insulin-like growth factor IGF-1 receptor [211]. High insulin levels induce cardiomyocyte hypertrophy through binding to IGF-1 receptors. IGF-1 produced by cardiomyocytes can also stimulate cardiomyocyte hypertrophy through ERK1/2 and PI3K signaling pathways [212]. The crosstalk pathway between IGF-1 and insulin signaling plays an important role in hyperglycemia/insulin resistance-induced cardiac hypertrophy and fibrosis in diabetic cardiomyopathy. IGF-1 knockdown reduces glucose uptake by the heart, increases cardiac reactive oxygen species (ROS) production, and induces mitochondrial dysfunction that impedes cardiac metabolism and function and increases fibrosis [213, 214]. Inflammatory factors such as TNF-*α*, NF-*κ*B, protein kinases, JNK, and P38 can directly induce myocardial hypertrophy and promote myocardial fibrosis [87, 215]. How Plin5 stimulates cellular hypertrophy and fibrosis through these inflammatory factors still needs further experimental exploration.

### 3.3. Microvascular Endothelial Dysfunction

Microangiopathy occurs widely in T2DM, leading to microcirculation abnormalities [216, 217]. Normal function of the coronary arteries and downstream microcirculatory vessels is impaired in diabetic cardiomyopathy. Structural abnormalities in the coronary microcirculation include luminal obstruction, inflammatory infiltration, vascular remodeling, and perivascular fibrosis [218]. Functional abnormalities in the coronary microcirculation include endothelial and smooth muscle cell dysfunction and impaired vasodilation and contraction as well as ischemia-reperfusion [218]. Coronary angiography suggests that microvascular endothelial dysfunction is an important cause of coronary microvascular disease (CMD) [219].

Microvascular endothelial cells are the basic structure of microvascular. Microvascular endothelial cells (CMECs) occur before DC cardiomyopathy [220] and are the earliest and most vulnerable target of heart damage from DM [221]. More than 67.1% of T2DM patients have abnormal lipid metabolism [222], and plasma FFAs gradually increase as early as two weeks before hyperglycemia [223]. Studies have shown that HFFA reduces the number of microvascular and destroys the integrity of microvascular in T2DM-HFFA mice [107]. Plin5 plays an important role in LD and FFA metabolism of CMECs, and its expression status determines its protective/destructive effect on CMECs.

Plin5 protects diabetic CMEC by bidirectionally regulating FFA metabolism through phosphorylated and nonphosphorylated states [102]. Nonphosphorylated Plin5 can inhibit lipolysis and promote mitochondrial contact of LDs, thus promoting FFA storage in LDs and reducing FFA damage to CMEC. Loss and phosphorylation of Plin5 can induce CMEC damage through oxidative stress as a potential mechanism [102]. Activation of PKA can stimulate phosphorylation of Plin5 [26, 224], and Ser155 may be an important site [26, 41]. Both the deficiency of Plin5 and phosphorylation pattern of Plin5 (P-Plin5) promote the hydrolysis of triglycerides (TG) in LD and the release of FAs to the cytoplasm [163]. In addition, P-Plin5 in the nucleus also inhibits SIRT1 activity, promotes PGC-1*α* transcription, and enhances mitochondrial synthesis and oxidation functions [41]. In physiological state, the phosphorylated and nonphosphorylated states of Plin5 can be transformed into each other and regulate the FFA content bidirectionally. When Plin5 phosphorylation or Plin5 deletion exceeds the body's ability to repair itself, excessive FFA and strong mitochondrial oxidation capacity will be generated, which will stimulate mitochondrial *β*-oxidation, produce a large number of ROS [225, 226], and reduce the expression and activity of endothelial nitric oxide synthase (eNOS) [227], ultimately leading to increased apoptosis rate, decreased capillary number, worsening structural insufficiency, and increased diastolic dysfunction [107].

## 4. Clinical Intervention

Controlling blood glucose level and lipid metabolism can effectively reduce blood glucose fluctuation and fatty degeneration in treatment of DC. Similar to the idea of hypoglycemia in diabetes, in order to reduce the damage of lipotoxicity on cells, clinical interventions can be considered to increase the storage of lipids to reduce the accumulation of excessive FFAs in the cytoplasm on the one hand and promote the oxidation level of fatty acids to accelerate the utilization of lipids to reduce steatosis on the other hand [228]. Based on the great potential of Plin5 in lipid regulation, we can interfere with the transcriptional expression of Plin5 and its involvement in lipolysis and LD-mitochondrial contact processes to achieve bidirectional regulation of FA metabolism.

### 4.1. Plin5/PPAR-*α*: Lipid Oxidation and Myocardial Hypertrophy

As previously known, PPAR-*α* regulates the transcriptional expression of Plin5 in the nucleus and plays a key role in Plin5-mediated lipid metabolism and mitochondrial oxidation. Activation of the PPAR-*α*-related signaling pathway directly affects the expression of key genes for FA oxidation [229]. Dapagliflozin, a member of the sodium-glucose cotransporter 2 inhibitors (SGLT-2), which is a new class of hypoglycemic agents, enhances renal excretion of excess glucose or glycerol [230]. Dapagliflozin (DAPA) has been widely used in the treatment of diabetes and cardiovascular disease [231–233]. DAPA mediated Plin5/PPAR-*α* signaling axis to reduce vascular endothelial growth factor-induced cardiac hypertrophy in vivo and in vitro [234, 235], while silting Plin5 can reverse the protective effect of this pathway [234]. At the same time, DAPA significantly improved cardiac function and increased ejection fraction [234–236]. It is important to note that DAPA had the same protective effect in both diabetic and nondiabetic patients, so the cardioprotective effect of DAPA was independent of the hypoglycemic effect of the drug [236, 237]. In conclusion, although SGLT-2 drugs act mainly in the kidney to promote the metabolism of excess glycolipids from the body, they can also interfere with the Plin5/PPAR-*α* pathway to regulate lipid metabolism and cardiomyocyte hypertrophy for cardiac benefit.

The absence of PPAR-*α* is known to lead to significant hypertrophic growth and cardiac dysfunction [238]. Experimental results showed that fenofibrate, as a PPAR-*α* agonist, can not only reduce cardiac hypertrophy by activating the PPAR-*α* signaling pathway that negatively regulates the binding activity of activated protein-1 (AP-1) [239, 240] but also significantly inhibit the activation of ERK1/2 and Akt induced by high glucose [241], reducing myocardial oxidative stress. Overexpression of PPAR-*α* targeting hypertrophic myocardium can also improve cardiac function by attenuating mitochondrial death pathways [242]. Therefore, fenofibrate has bright clinical benefits as PPAR-*α* receptor agonists in improving lipid-induced cardiac hypertrophy and cardiac dysfunction.

### 4.2. GPR40: FFA Receptor, Bidirectional Regulation of Insulin

The GPR40 agonists are also expected to be a novel intervention drug for DC based on the mechanism of insulin resistance/deficiency pathway between Plin5 and DC. GPR40 agonists are currently being used as a novel treatment for type 2 diabetes [243]. GPR40, also known as FFA receptor 1 [244], activates phospholipase production of diacylglycerol, which increases insulin secretion [244–246]. Clinical trials have shown that the GPR40 agonist TAK-875 can reduce fasting and postprandial blood sugar and the HbA1c without causing hypoglycemia or lipid toxicity [247–250]. GPR40 agonist can promote insulin secretion and hypoglycemia without causing lipid toxicity, showing unique advantages in regulating glucose and lipid metabolism, which has great research and development potential.

Some foods may also enhance insulin sensitivity through the Plin5-related pathway. For example, sweet potato squash improves insulin sensitivity through PPAR-*γ* and GLUT4 that activates 3T3-L1 adipocyte IRS-1/PI3K/AKT pathway [251]. Promoting insulin secretion and increasing insulin sensitivity is a relatively ideal treatment direction for insulin resistance/deficiency lipid metabolism abnormalities, which can effectively accelerate the utilization or storage of FAs to reduce lipid toxicity. As an important target for improving insulin resistance/deficiency, Plin5 provides new ideas for future treatment of DC.

### 4.3. Other Mechanisms and Interventions

Research shows that Plin5 ameliorates podocyte injury induced by high glucose by inhibiting Akt/GSK-3BETA/NRF2-mediated apoptotic oxidative stress and inflammation [252]. The result suggests that intervention of Plin5-related inflammatory and oxidative stress pathway may also reduce DC. For example, allopurinol can activate Nrf2/P62 and reduce oxidative stress, thereby alleviating diabetic cardiomyopathy in rats [253]. Resveratrol alleviates diabetic cardiomyopathy in rats by improving mitochondrial function through PGC-1*α* deacetylation [254].

Because of the complex interactions between various mechanisms such as oxidative stress, inflammation, and IR, the process by which Plin5 regulates lipid metabolism is also intricate. Therefore, the pathway of clinical interventions to regulate lipid metabolism by Plin5 is not as homogeneous as presented by purely experimental data. Nevertheless, this does not negate the importance of Plin5 in DC lipid metabolism and the effectiveness of current clinical interventions.

## 5. Conclusions

Lipid toxicity caused by DC metabolism disorder can aggravate myocardial cell injury, promoting heart failure, ventricular remodeling, cardiac dysfunction, and other adverse events. As the pathogenesis of DC is complex, the clinical application of RAAS system inhibitors and *β*-blockers is not effective, and it is very important to find new DC intervention targets. Plin5, as a LD-associated protein, is highly expressed in the heart and is closely related to energy metabolism-related organelles such as the nucleus, mitochondria, and ER, playing an irreplaceable role in regulating lipid metabolism.

The role of Plin5 in lipid metabolism in cells has been reported. Plin5 participates in the synthesis of TG by FAs in the ER, forming LDs and reducing the lipid toxic damage caused by excessive FFAs in cells. When stimulated, Plin5 binds to ATGL and HSL to promote lipolysis of LD and releases FAs. The released FAs can be transported to mitochondria through Plin5-mediated LD-mitochondrial contact for FA *β*-oxidation to release energy, thus reducing excessive accumulation of FFAs in cells.

Plin5 can multidimensionally protect the body damaged by lipid toxic of DC, such as regulating lipid balance, reducing oxidative stress and inflammation levels, reducing IR and ER stress, protecting mitochondria and endothelial cells, and delaying apoptosis. On the one hand, Plin5 regulates oxidative stress by activating the PI3K/Akt pathway to regulate mitochondrial proliferation and apoptosis. Plin5, on the other hand, mediates cell metabolism and apoptosis by interfering with the MAPK pathways (P38, ERK, and JNK), including mediating the inflammatory response by inhibiting the P38 pathway, promoting cell proliferation by activating the ERK pathway, and promoting insulin receptor signaling by inhibiting the JNK pathway. ER stress is closely related to insulin resistance, and the specific mechanism is still unclear. It may be related to lipid toxicity of islet *β*-cells caused by overnutrition. Plin5 can also improve mitochondrial metabolism through the PPAR-*α*/PGC-1*α* signaling pathway and prevent the mitochondrial dysfunction caused by it. Each signaling pathway is different, but each signaling molecule is closely related and interacts with each other, jointly improving the pathological changes caused by DC lipid toxicity under the action of Plin5.

Fortunately, there are some novel drugs for Plin5 to intervene in DC lipotoxic injury, such as SGLT-2 inhibitor (DAPA), PPAR-*α* agonist, and GPR40 agonist. These drugs not only boost metabolism but also protect the cardiovascular system. Studies have shown that they can improve antioxidant, anti-inflammatory, and insulin resistance to some extent, reduce lipid toxicity, and protect the myocardial cell without causing hypoglycemia.

However, despite the fact that relevant experiments have proven clinical benefit of Plin5, a large number of experiments and data are needed for further verification. In addition, Most of the conclusions are based on animal experiments, and clinical observation is relatively scarce. Therefore, in future studies, (a) broadening the network of Plin5 targets and cofactors at different stages of DC pathogenesis will help to determine the more specific role of Plin5, possibly using the protein as a tool for diagnostic or therapeutic targeting. (b) In terms of therapy, a comprehensive understanding of the mechanism of Plin5 DC protection and its related signaling pathway may be of additional value to overcome the limitations of a single measure and target. (c) On the basis of more abundant and mature laboratory studies, more clinical studies should be carried out appropriately.

## Figures and Tables

**Table 1:** Physiological effects and related mechanisms of Plin5.

Tissue	Model	Findings	Reference
Heart	Plin5^−/−^ mice	↑ cardiac FA oxidation ↓ diacylglycerol and ceramide ↓ LDs and TAG content ↑ heart weight No clear effect on glucose oxidation and sensitivity to hypoxia	[46, 108, 109]
	Plin5^+/+^ mice	No clear effect on glucose oxidation in isolated cardiomyocytes	[108]
	CM-Plin5 mice	↑ TAG content ↑ TAG hydrolytic activities: ↑ ATGL and CGI-58 protein levels ↓ FA oxidizing gene expression levels	[72]
	MCK-Plin5 mice	↑ TAG content	[73]
	MKO mice	↓ TAG content No ER stress, inflammation, and oxidative stress	[110]

Liver	Plin5^−/−^ mice	↓ hepatic TAG content in fed state ↑ TAG content in fasting state ↑ lipolysis ↑ mitochondrial proliferation ↑ mitochondrial oxidative capacity ↑ expression of proinflammatory genes under an HFD ↑ expression of ER stress-related genes ↑ lipid peroxidation ↑ insulin sensitivity	[24, 35, 70]
	MCK-Plin5 mice	↓ lipid uptake ↓ inflammatory markers	[73]
	Hepatocyte-specific Plin5^−/−^ mice	↓ FA consumption and FA oxidation ↓ TAG secretion ↓ lipid peroxidation and oxidative stress ↑ insulin resistance under an HFD ↑ glucose intolerance under HFD ↑ TAG accumulation under HFD	[111]
	Plin5^+/+^ hepatocytes cell	↑ hepatic TAG accumulation and hepatic steatosis ↓ VLDL content	[112]

Vessel	Plin5^−/−^ mice	↓ LD content ↑ the level of intracellular FFAs in CMECs ↓ NO released in CMECs ↑ ROS generation ↓ the number and structural of cardiac microvascular ↑ apoptosis rate of CMECs and diastolic dysfunction	[113]
	MCK-Plin5 mice	↑ LDs and intramyocellular TAG ↓ body weigh ↑ oxygen consumption ↑ muscle ER stress No obvious defects in mitochondrial	[73]

Pancreas	Plin5^−/−^ mice	↑ apoptosis ↑ palmitate-mediated mitochondrial dysfunction No augment lipotoxicity	[32]
	Plin5^+/+^ mice	↓ palmitate-mediated mitochondrial dysfunction ↓ oxidative stress ↑ antiapoptotic	

BAT	Plin5^−/−^ mice	↓ mitochondrial respiration	[34]
	Plin5^+/+^ mice	↑ glucose tolerance and insulin sensitivity at room temperature ↓ weight during cold exposure	

WAT	Plin5^+/+^ mice	↑ insulin sensitivity ↓ inflammation	[34]
Muscle	Plin5^−/−^ mice	↑ skeletal muscle insulin resistance ↓ TAG content	[24, 70, 114]
	MKO mice	↑ fat mass ↓ respiratory exchange ratio ↑ FA oxidation under HFD ↑ oxidative stress ↑ TAG content ↓ proinflammatory markers	[110]
	MCK-Plin5 mice	↑ LD formation ↓ body weight compared to nontransgenic littermates under control and HFD diet ↑ expression of ER stress markers	[73]

Plin5^−/−^: Plin5 deficient; Plin5^+/+^: Plin5 overexpression; FA: fatty acid; LD: lipid droplet; TAG: triacylglyceride; CM-Plin5: cardiac muscle-specific overexpression of Plin5; ATGL: adipose triglyceride lipase; CGI-58: comparative gene identification-58; MCK-Plin5: skeletal muscle-specific overexpression of Plin5; MKO: muscle-specific Plin5 knockout; ER: endoplasmic reticulum; HFD: high-fat diet; VLDL: very-low-density lipoprotein; CMECs: cardiac microvascular endothelial cells; FFAs: free fatty acids; BAT: brown adipose tissue; WAT: white adipose tissue; ROS: reactive oxygen species.
